# A comparative study of four Bowie-Dick test under the condition of pressure steam sterilizer simulating gas leakage

**DOI:** 10.1097/MD.0000000000023653

**Published:** 2020-12-11

**Authors:** Xiaomei Yu, Qing Zhang, Xingling Deng, Jinxiu Liang, Shuqin Hao, Jianling Chen, Heying Du

**Affiliations:** a Disinfection Supply Center, The First Affiliated Hospital of Sun Yat-sen University, Guangzhou; b Disinfection Supply Center, Peking Union Medical College Hospital, Chinese Academy of Medical Sciences, Beijing; c Disinfection Supply Center, Affiliated Ophthalmic Hospital of Sun Yat sen University, Guangzhou; d Disinfection Supply Center, Shenzhen Second People's Hospital, Shenzhen, P.R. China.

**Keywords:** Bowie-Dick test, gas leakage, pressure steam sterilizer

## Abstract

We aimed to understand the evaluation of different Bowie-Dick test (B-D test) on the performance of pressure steam sterilization equipment in the case of simulated gas leakage, and we selected a pulsating vacuum steam sterilizer to set 4 different gas leakage levels: 1.1, 1.3, 1.5, and 1.7 mbar/min during the B-D test phase. In terms of methods, 4 different brands of B-D test kits (devices) were tested at 4 different leakage rates, and a total of 48 experiments were conducted. The results from univariate analysis revealed that there are statistically significant differences in the judgment of test results among different personnel and brands. The results from multivariate logistic regression analysis displayed that the difference between different personnel was statistically significant (*χ*^2^ = 45.34, *P* < .001); the difference between different products was statistically significant (*χ*^2^ = 129.37, *P* < .001); and there was no statistically significant difference between different degree of leakage (*χ*^2^ = 6.99, *P* > .05). Result judgments of brand 1 and brand 2 are susceptible to subjective factors. The judgment of brand 3 is intuitive and consistent with the evaluation result of brand 4. In conclusion, the order of capacity to evaluate air leakage from best to worst is brand 4→brand 3→brand 1→brand 2.

## 1 Introduction

The presence of air pocket is the main factor causing the failure of sterilization.^[1,2]^ Therefore, the prevacuum (including pulsating vacuum) pressure steam sterilizer should perform the Bowie-Dick test (B-D test) without load before the start of sterilization operation every day. The successful air removal in a sterilizer chamber is qualified for the next procedure.^[3]^ The B-D test can be applied to evaluate the success of a sterilizer with porous load sterilization to remove air.^[4]^ At present, B-D test is mainly divided into chemical and physical approaches. The chemical B-D test is mainly used in China, whereas the physical electronic B-D test is mainly used in foreign hospital disinfection supply centers, but not in China.^[5,6]^ At present, the technical requirement of large-scale steam sterilizer is that the pressure rising rate required by vacuum leakage test should not exceed 0.13 kPa/min (1.3 mbar/min).^[7]^ However, whether the daily clinical B-D test can assess an inadequate vacuum has not been clarified.

In this study, 4 different leakage rates were set by simulating gas leakage of pressure steam sterilizer, and B-D test results of 4 different brands were judged in the presence of cold air. By the contrast of 4 different brands of B-D test results, we might reveal the reliability evaluation method for the steam sterilizer by rational utilization of the B-D test.

## 2 Materials and methods

### 2.1 Pressure steam sterilizer

In April 2019, an AG steam sterilizer [Belimed, MST-H 9-6-9HS2 (No. 6)] was used in the First Affiliated Hospital of Sun Yat-sen University, Guangzhou 510080, China. A microleakage valve device and a gas flow meter device were installed on the test connector of the sterilizer. With the valve connecting to the chamber, we could manually control the outside air injection into the chamber through the valve switching during the sterilization process. Four brands of B-D test included brand 1 (Imported Chemistry), brand 2 (Domestic Chemicals), brand 3 (Imported Tubes), and brand 4 (Imported Electronics) were used. Each brand was the same batch. Parameters of B-D test program were temperature 134°C (3.3 min).

### 2.2 Experimental measurement

Before the simulated gas leak experiment, 3 pre-experiments were carried out under the condition that the leakage rate of sterilizer was 0 mbar/min. The results of the 4 brands were all qualified. P6 leak test program in the device was run, and the batch record was checked after the program ended. The leak rate was 0 mbar/min. The microvalve of the leakage device connected with the chamber was open, and the P6 leak test program was run again. The value of the air flow entering the chamber could be observed by the leakage device equipped with a gas flow meter to adjust the microvalve. After the program was completed, the leak results recorded by the batch were checked. Finally, the 4 leak rates were 1.1, 1.3, 1.5, and 1.7 mbar/min. P5 B-D test program with B-D test pack (device) were run under different leak rates obtained above, and the leak valve was opened during entire process. At 4 gas leakage levels, the 4 B-D test products were grouped according to the Latin variance design. Each was tested 3 times at the 4 different leak rates.

### 2.3 Judgment of results

The chamber door was opened, the B-D test pack (device) was taken out, and the result was assessed according to the product specification. For the brand 1, brand 2 B-D test paper pattern color all changed to uniform black was judged qualified. For brand 3 B-D test, all 6 color zones were converted to black, indicating that the test was qualified. For brand 4 B-D test, the electronic B-D device was started by the software, and then the electronic B-D device was put into the sterilizer chamber to run the B-D program. After the program was finished, the electronic B-D device was put into the software to read the data, and the system could automatically judge whether the test was qualified according to the temperature and pressure detected by the electronic device. Finally, the test results were arranged by a random number table, and the test results were judged by 7 sterilizers and 14 nurses (central sterile supply department professional years were >3 years), and the judges were blinded.

### 2.4 Statistical analysis

The data were analyzed using Statistical Product and Service Solutions 22.0 statistical software with the adoption of binary category logistic regression analysis (input method). The results of 4 different B-D test products under 4 different leak rates were compared. The comparison between different leak rates was counted using *χ*^2^ test or Fisher Exact Test. *P* < .05 was considered to be statistically significant. The pairwise comparison was revised according to the Bonferroni method; the test level was 0.05/6 = 0.0083, and *P* < .0083 was considered statistically significant.

## 3 Results

### 3.1 Univariate analysis of the judgment results of different personnel, leak rates, and B-D test products

The judgment of different personnel on the test results was that the qualified rate of sterilizer was 33.3%, and that of nurses was 73.2%. The difference between different judges was statistically significant, *χ*^2^ = 4.26, *P* < .001.

There was no significant difference between the test results of 4 leakage rates with different leakage rates, *χ*^2^ = 1.72, *P* > .05.

The test results of different brands were judged to be statistically significant, *χ*^2^ = 729.97, *P* < .001. Pairwise comparison was described as follows: the qualification rate of brand 2 (91.7%) was higher than that of brand 1 (74.2%), *χ*^2^ = 27.14, *P* < .001; the qualification rate of brand 3 and brand 4 (0.0%) was lower than that of brand 1 (74.6%), *χ*^2^ = 297.31, *P* < .001; the qualification rate of brand 3 and brand 4 (0.0%) was lower than that of brand 2, *χ*^2^ = 426.46, *P* < .001; and the order of evaluating leakage capacity from good to bad was brand 4→ brand 3→ brand 1→ brand 2 (Table 1).

**Table 1:** Univariate analysis of the judgment results (n = 1008).

Variables	Level	Qualified number	Ratio of qualification (%)	*χ*^2^	*P*
Personnel	1	112	33.3	4.26	.001
	2	306	73.2		
Product	1 (Brand 1)	187	74.2		
	2 (Brand 2)	231	91.7^∗^	27.14	.001
	3 (Brand 3)	0	0^†^^,^^‡^		.001
	4 (Brand 4)	0	0^†^^,^^‡^		.001
Leak rate	1.1 mbar/min				
	1.3 mbar/min			1.72	.639
	1.5 mbar/min				
	1.7 mbar/min				

∗ Brand 1 comparing brand 2, *χ*^2^ = 27.14, *P* < .001.

† Brand 3 and brand 4 comparing brand 1, *χ*^2^ = 297.31, *P* < .001.

‡ Brand 3 and brand 4 comparing brand 2, *χ*^2^ = 426.46, *P* < .001.

### 3.2 Multivariate logistic regression analysis of judgment results of different personnel, product, and leak rate

The influence of mutual factors on the judgment of test results was corrected. The statistical regression equation contained variables of different personnel and brands. The difference between different people was statistically significant, *χ*^2^ = 45.34, *P* < .001; the difference between brands was also statistically significant, *χ*^2^ = 129.37, *P* < .001; and there was no significant difference between different leakage degrees, *χ*^2^ = 6.99, *P* > .05 (Table 2).

**Table 2:** Multivariate logistic regression analysis of judgment results (n = 1008).

							95% Confidence interval	
Variables	B	SE	Wald	df	Sig	Exp (B)	Upper limit	Lower limit
Personnel (1: control group)	−1.797	0.267	45.342	1	0.000	0.166		
Leak rate			6.988	3	0.072	0.295		
Brand			129.374	3	0.000			
Brand 4 (control group)						1.000		
Brand 1	7.102	1.024	48.086	1	0.000	1214.610	163.172	9041.265
Brand 2	8.620	1.047	67.756	1	0.000	5541.903	711.645	43,157.340
Brand 3	0.000	1.418	0.000	1	1.000	1.000	0.062	16.117
Constant	−5.537	1.032	28.778	1	0.000	0.004		

## 4 Discussion

Due to equipment problems, such as the reduced efficiency of the vacuum pump which caused the incomplete removal of air in the sterilizer cabinet, and the leaking seal washer of the door which caused air remaining in the sterilizer cabinet. The residual air could prevent the penetration of steam to the medical device during the steam exposure phase of the sterilization process. The noncondensable gas brought into the steam might make the temperature in the articles unable to reach the set temperature value, affecting the sterilization effect.^[8]^ It has been reported that high atmospheric pressure pulses can be used to improve the B-D test and biological test conformity by changing the pulsating vacuum method.^[9]^ In these experiments, 4 different leakage levels were artificially set by simulating air leakage in the sterilizer, and our test was executed by adopting 3 chemical B-D test product (devices) and a latest electronic B-D in clinic. The results exhibited that under the same B-D test procedure, the test results of the same brand were judged by the sterilizer and the nurse, respectively, and the results judged by the 2 were inconsistent. In terms of ability to judge a gas leak as substandard, the sterilizer was superior to the nurse, suggesting that sterilizer operator has the ability to find problems after a long period of professional theoretical and operational training.

In previous study, some scholars compared the performance of 3 kinds of B-D test products in normal use, and found that the qualification rate of the one-time B-D (Xinhua) test package was 99.2% and that of the GKE lumen simulation device was 97.5%, and there was no statistical difference between the 2.^[10]^ In this study, the results from univariate and multivariate analyses showed that there were statistically significant differences between the test results of the 4 brands under the same B-D test procedure. When the gas leakage rate was between 1.1 and 1.7 mbar/min, the qualification rates of brand 1, brand 2, brand 3, and brand 4 were 74.2%, 91.7%, 0%, and 0%, respectively.

B-D test papers of brand 1 and brand 2 are made of thermosensitive dye in a Chinese character “meter” profile, when the color changed to uniform black, the result is qualified; when the discoloration is uneven but typical, the result could be intuitively judged. However, when the discoloration is not typical, it is susceptible to subjective factors because there is no quantitative observation index. In addition, the Chinese character “meter” has a small black and white angle in the middle, and the contrast is relatively weak, which can affect the judgment of the uniformity degree, and a “false negative or false positive” results will appear. Moreover, when the air leakage rate is between 1.1 and 1.7 mbar/min, as the air leakage rate increases, the failure rate of the brand 1 and brand 2 B-D is not increasing. Therefore, the result judgment cannot objectively reflect the actual situation of the sterilizer under the air leakage.

The B-D device of brand 3 consists of a lumen structure with chemical indicator that simulates the original B-D test, and the lumen simulator is more sensitive to cold air remains or leaks. The B-D device of brand 3 can instantly react to 1 mL noncondensable gas. When all the 6 color zones of the chemical indicator in the lumen simulation device are turned into black, the steam penetration is completed. As the temperature and time reach the sterilization requirement, the longer the time, the darker the color. Pairwise comparison of brand 3 with brand 1 and brand 2 showed statistically significant differences. Under the gas leakage rate of 1.1 to 1.7 mbar/min, only 2 to 4 of the 6 color zones were converted to black, so the test results were judged to be unqualified, and the intuitiveness was very good.

The B-D device of brand 4 belongs to the physical monitoring but is different from the results of physical monitoring with the temperature probe of the sterilizer. It has a lumen type B-D device. Compared with artificial chemical monitoring and biological monitoring, the real-time electronic B-D can dynamically and comprehensively reflect the physical properties and sterilization effect of the sterilizer, and objectively reflect whether the key parameters meet the setting requirements.^[11]^ The electronic B-D is a data recorder with 2 temperature sensors and a pressure sensor, which, as an electronic replacement for chemical indicators, can perform B-D test and batch process control daemon. Under the gas leakage rate of 1.1 to 1.7 mbar/min, the temperature in electronic B-D recorder was lower than the set temperature in some periods, and the test results could be directly determined. In addition, the results were consistent with brand 3, and were not affected by personnel or multiple judgments. It also could objectively reflect the existence of gas leakage in the sterilizer, and provide early warning for unqualified sterilizers.

We proved that under the simulated sterilizer gas leakage, the judgment results of different personnel were inconsistent under the same B-D test procedure; the different B-D test packs (devices) had different evaluations on the performance of the sterilizer; no quantitative index could be used for the judgments in the B-D test product of brand 1 and brand 2, and thus the prediction was susceptible to subjective factors; the judgment of the B-D test product in brand 3 without quantitative indicators was intuitive and accurate; the results were consistent for B-D evaluation results of brand 3 and brand 4; and the electronic B-D test system could objectively and quantitatively evaluate the key physical parameters and the effectiveness of the steam sterilizer during sterilization process. It also could timely find the air leakage fault of the sterilizer to ensure the safe operation of the sterilizer, and effectively guarantee the quality of aseptic supplies.

## Author contributions

**Data curation:** Xiaomei Yu, Jianling Chen.

**Formal analysis:** Jinxiu Liang.

**Software:** Shuqin Hao.

**Writing – original draft:** Xiaomei Yu, Qing Zhang, Xingling Deng.

**Writing – review & editing:** Heying Du.
